# Effects of the menstrual cycle on the performance of female football players. A systematic review

**DOI:** 10.3389/fphys.2024.1359953

**Published:** 2024-04-08

**Authors:** Dina Hamed-Hamed, Ana González-Muñoz, Maria Cuevas-Cervera, Jose Javier Perez-Montilla, Daniel Aguilar-Nuñez, María Aguilar-García, Leo Pruimboom, Santiago Navarro-Ledesma

**Affiliations:** ^1^ Clinical Medicine and Public Health PhD Program, Faculty of Health Sciences, University of Granada, Granada, Spain; ^2^ Clinica Ana Gonzalez, Malaga, Spain; ^3^ Department of Nursing and Podiatry, Faculty of Health Sciences, University of Malaga, Malaga, Spain; ^4^ Biomedicine PhD Program, Faculty of Health Sciences, University of Granada, Granada, Spain; ^5^ University Chair in Clinical Psychoneuroimmunology, University of Granada and PNI Europe, Melilla, Spain; ^6^ Department of Physical Therapy, Faculty of Health Sciences, University of Granada, Melilla, Spain

**Keywords:** menstrual cycle, luteal phase, women football, follicular phase, performance, psychosocial factors

## Abstract

**Background:**

Women’s football has been booming for a few years now, which has led to an increase in the expectation of the players’ performance, leading to a more detailed study of women’s physiology in the field of sports.

**Objectives:**

To analyze the scientific evidence on the influence of menstruation on the performance of female footballers, as well as to analyze the methodological quality of the studies included in this review.

**Materials and methods:**

The possible hormonal effects of the menstrual cycle phases on the performance of female footballers were analyzed. The databases used to conduct the searches were Pubmed, Scopus, Virtual Health Library, Web of Science, EBSCO and the Cochrane Library. All included studies met the inclusion criteria. The Cochrane risk of bias tool was used. This systematic review protocol was registered at the International Prospective Register of Systematic Reviews (PROSPERO: CRD42023390652).

**Results:**

A total of nine clinical trials were included in this review. A low quality of evidence was observed in the studies. Not all the results support the idea that the menstrual cycle phases can alter the performance of female footballers.

**Conclusion:**

This systematic review shows that there is a great deal of controversy about the influence of the menstrual cycle phases on the performance of female footballers. Studies are focused on solely biological factors and gender is normally no part of those studies. Further research with larger samples, and taking not only biological but also sociological factors, are necessary to determine the effects of menstruation on the performance of female footballers.

## 1 Background

In recent years, women’s football has developed exponentially (López-Valenciano et al., 2021). At the European level, the number of women registered as players with the Union of European Football Associations (UEFA) rose from 1.27 million in 2016 to 1.35 million in 2017 (Easter and Perry, 2014; Alahmad et al., 2020), with the UEFA financial support tripling and participation rates in recent years up by a third (Randell et al., 2021). There are currently more than 30 elite national women’s football leagues established mainly in the following European countries: Sweden, France, Spain and Germany (López-Valenciano et al., 2021).

The current FIFA President reflected on the growing popularity of women’s football around the world and highlighted the clear objective of continuing to support its growth with the following statement: “The future of football is women’s” (Martínez-Lagunas et al., 2014). Currently, some 29 million women play football, which corresponds to 10% of the total number of footballers worldwide (Martínez-Lagunas et al., 2014). The number of international competitions has also increased, with athletes having more opportunities to practice this sport (Martínez-Lagunas et al., 2014).

It was once thought that the physiology of exercise was the same for both sexes, so the recommendations for sports practice in women have been widespread for a decade (García-Pinillos et al., 2021). With women’s sport booming, the expectations of female players’ performance have increased and there has been a need to improve physiological knowledge of exercise in women, thus increasing the need for specific scientific research that can help in their performance (Martínez-Lagunas et al., 2014; García-Pinillos et al., 2021). Nowadays, players are exposed to a greater amount of training and demand, therefore, it is necessary to understand the physiology of the players in order to design adequate training (Pardos-Mainer et al., 2021). Physiology is not only influenced by biological factors related with sex; sociological factors ‘creating’ gender show equal or even more influence on performance than biology (Hallam et al., 2022). Gender is still a widely neglected factor in all levels of human society and especially in sports. The binary division between men and women has produced many studies with biological bias, neglecting the impact of sociological factors, including social expectations about the way men and women ‘should behave’ (Hentschel et al., 2019).

Our systematic review focused on the impact of the menstrual cycle on performance in women football. We predicted to find important effects of different hormones on women performance during the menstrual cycle and chose this topic because of the supposed isolated biological influence of sex hormones in women.

Football in general is a sport that requires motor, technical, tactical and psychological skills, therefore, it is interesting to observe its impact on the musculoskeletal and neuroendocrine systems, and in the case of women, the influence of menstrual cycle hormonal fluctuations on female footballers during their games (Foster et al., 2017). A soccer match lasts approximately 90 min, requiring both aerobic and anaerobic capacities. Professional players typically cover around 10 km during a match, engaging in numerous explosive activities such as jumps, sprints, kicks, changes of pace, and direction shifts, utilizing 80%–90% of their maximum heart rate (Stølen et al., 2005). Therefore, there are studies indicating that the menstrual cycle should be taken into account to optimize the performance of female players (Forsyth et al., 2023).

There are several studies that link physical activity with menstrual changes, but only a few evaluate the influence of menstrual cycle phases on sports performance (Foster et al., 2017).

It should be emphasized that the menstrual cycle is perhaps the second most important biological rhythm after the circadian one (Constantini et al., 2005) and causes large variations in reproductive hormones that could hypothetically influence football performance through the direct effects of these hormones (Randell et al., 2021). The menstrual cycle starts around 11–13 years of age and ends with menopause around the age of 50 (Reilly, 2000). This cycle is the result of the action of hypothalamic, pituitary and ovarian hormones that cause various changes in the female reproductive system and in many other tissues (Cable and Elliott, 2004). Although estrogen and progesterone are the main hormones, the effects of other hormones such as testosterone, relaxin, and leptin have also been explored (Constantini et al., 2005).

The cycle is composed of the hypothalamic-pituitary-ovarian axis, with the secretion of gonadotropin-releasing hormone (GnRH) stimulating the release of follicle-stimulating hormone (FSH) and luteinizing hormone (LH) which signal the ovaries to synthesize estrogen and progesterone (Cable and Elliott, 2004; Brown et al., 2021). A normal menstrual cycle lasts about 28 days, and consists of a follicular phase typically lasting 12–14 days where estrogen levels are high and progesterone levels are low, an ovulatory phase that lasts 1–3 days with a rise in estrogen levels and a luteal phase that lasts 12–14 days with high levels of progesterone and medium levels of estrogen (Draper et al., 2018; Dirk et al., 2020; García-Pinillos et al., 2021; Paludo et al., 2022). It has been demonstrated that estrogen can influence the cardiovascular system (including blood pressure, heart rate and rhythm, and vascular flow) as well as substrate metabolism (Constantini et al., 2005), in practice, elevated estrogen levels are associated with lower blood lactate levels and longer times to exhaustion (García-Pinillos et al., 2021). Estrogen is a trigger for the release of neurotransmitters that attenuates the release of aminobutyric acid, which is a neurotransmitter responsible for reducing muscle tone (Carmichael et al., 2021). Dehydroepiandrosterone (DHEA), estrogen and testosterone produce an excitatory effect. Variations in testosterone throughout the menstrual cycle produce physiological effects that can alter strength; hence the performance of athletes can be disturbed (Blagrove et al., 2020). Progesterone is known to have a sympathetic effect, increasing heart rate, body and basal temperatures, and respiration; this effect plays a key role during exercise as it appears to increase perceived exertion and decreases athletic performance, especially in hot and humid environments. (García-Pinillos et al., 2021).

We observed that 15 international rugby players participated in a study on the influence of the menstrual cycle on performance, where 67% of the athletes considered that symptoms related to the menstrual cycle affected their performance, while 33% perceived heavy menstrual bleeding. Two-thirds of the athletes self-medicated to alleviate the symptoms (Findlay et al., 2020). However, there is still no consensus on the influence of hormonal variations induced by the phases of the menstrual cycle on physical performance and some studies report that no effect occurs (García-Pinillos et al., 2021).

In addition, it has been recognized that the menstrual cycle causes affective, physical and behavioral symptoms that develop during the luteal phase and disappear within a few days of menstruation. Menstruation causes symptoms such as cramps, low back pain and swelling in athletes, which could be directly related to the decrease in performance observed in training during these days (Brown et al., 2021), which is consistent with the results of another study that found that pain affects performance (Kishali et al., 2006) with menstrual cramps being associated with increased prostaglandin hormone production (Kishali et al., 2006).

It has been observed that dysfunctions of the menstrual cycle such as heavy bleeding affect the performance of athletes. Not all women and their cycles are the same, so it is important to take into account the duration of the cycle, bleeding pattern and symptoms individually (Bruinvels et al., 2022). Additionally, performance is thought to be worse in the early follicular phase because menstrual blood loss is a leading cause of iron deficiency anemia. Iron is an essential component of hemoglobin in red blood cells, which transports oxygen to the muscles. If this is deficient, an athlete cannot effectively utilize her aerobic capacities (Bruinvels et al., 2016). Much of the published research does not take into account the affectation of the menstrual cycle symptoms in female athletes (Kishali et al., 2006). Therefore, analyzing the current literature on the influence of menstruation on women’s football is of great relevance and interest.

The aim of this review is to analyze the scientific evidence on the influence of menstruation on the performance of female footballers, as well as to analyse the methodological quality of the studies included in this review.

This review formulates the following hypothesis: If during the menstrual cycle players experience significant hormonal fluctuations, these variations could hypothetically influence football performance. This hypothesis is supported by two studies: in one of them, 77% of elite athletes report adverse effects of the menstrual cycle (Martin et al., 2018), and in the second study, it is suggested that during the mid-luteal phase, when estrogen and progesterone concentrations are high, there is a decrease in performance, in factors such as endurance (Janse de Jonge, 2003).

## 2 Material and method

### 2.1 Study design

A systematic review, in which clinical studies were included, was carried out following the recommendations of the Preferred Reporting Items for Systematic Review and Meta-Analysis (PRISMA) (Welch et al., 2012) standard and followed the PICO strategy. The systematic review protocol was registered at the International Prospective Register of Systematic Reviews (PROSPERO: CRD42023390652). The objective of this search was to find scientific evidence on how menstruation affects the performance of female football players.

### 2.2 Documentary sources consulted

The following computerized databases were consulted: Pubmed, Cochrane Library, Virtual Health Library, EBSCO, Scopus and Web of Science.

### 2.3 Search strategy

To develop the search strategy, keywords extracted from the Medical Subject Headings (MesH) thesaurus were used, namely: “Menstrual Cycle”, “Luteal Phase”, “Follicular Phase” and “Athletic Performance”. The following terms that were not obtained from the MesH thesaurus were also used: “Female Soccer Players”, “Period” and “Performance”. These terms were combined with the Boolean operators AND and OR. The terms had to appear in the title, abstract and keywords.

The last search was carried out on 02/02/2024.

Supplementary Table S1 shows the search strategies.

### 2.4 Inclusion criteria

Clinical trials that discussed the effect of menstruation on female soccer players were included.

### 2.5 Exclusion criteria

The studies where players had suffered an injury within a 3-month period before starting the study were excluded.

### 2.6 Study selection process

Firstly, using the Rayyan QCRl programme, (Ouzzani et al., 2016), duplicate studies that were found in the different databases were removed. Next, the study selection process was carried out through a selective reading of the title and abstract. Subsequently, a full-text reading of the articles that apparently met the inclusion criteria took place.

The selection process was carried out on an individual basis.

### 2.7 Data extraction

The PICO strategy was used to extract data such as the general characteristics of the studies and their sample (author, year of publication, type of study design and place where the study was conducted, sample size, sample losses, age, sex, height, body mass and professional category of the players), characteristics of the intervention (type of intervention, duration, variable measure, measurement instrument) and the main results of the intervention.

### 2.8 Risk of bias measurement tool

The Cochrane Handbook of systematic reviews of interventions was used to assess the risk of bias of the included studies (Higgins and Green, 2011). This tool assesses seven domains, where each domain is evaluated with three possibilities: “High risk” (−), “low risk” (+) and “unclear risk” (?). The domains that were used for risk of bias assessment were the following: selection bias, performance bias, detection bias, attrition bias, reporting biases and finally other sources of bias where there was the opportunity to point out unaddressed biases that were considered important.

### 2.9 Quality of the evidence

The Grading of Recommendations, Assessments, Development and Evaluation (GRADE) (Aguayo-Albasini et al., 2014) system was the tool used to assess the quality of the evidence for the results reported by the studies (MENSTRUAL, 2009; Tsampoukos et al., 2010; Julian et al., 2017; Vila, 2017; Tounsi et al., 2018; Carmichael et al., 2021; Nabo et al., 2021; Campa et al., 2022; Igonin et al., 2022). This system defines the quality of the evidence as the level of confidence that we have on the estimation of an effect being adequate to make a recommendation. Assessment of the evidence quality includes risk of bias in the studies, inconsistency, imprecision, publication bias, proxy results and other factors that may influence the quality of the evidence.

## 3 Results

### 3.1 Study identification and selection process

In the process of identifying and selecting articles, a total of 317 articles were located in the different computerized databases. After eliminating the duplicates, the title and abstract of 64 studies were read to assess whether the selected articles met the inclusion criteria. A total of 9 articles met these criteria and the full texts were evaluated.

Finally, after fully reading the texts, 9 studies (MENSTRUAL, 2009; Tsampoukos et al., 2010; Julian et al., 2017; Vila, 2017; Tounsi et al., 2018; Carmichael et al., 2021; Nabo et al., 2021; Campa et al., 2022; Igonin et al., 2022) formed part of this systematic review.

**FIGURE 1:**
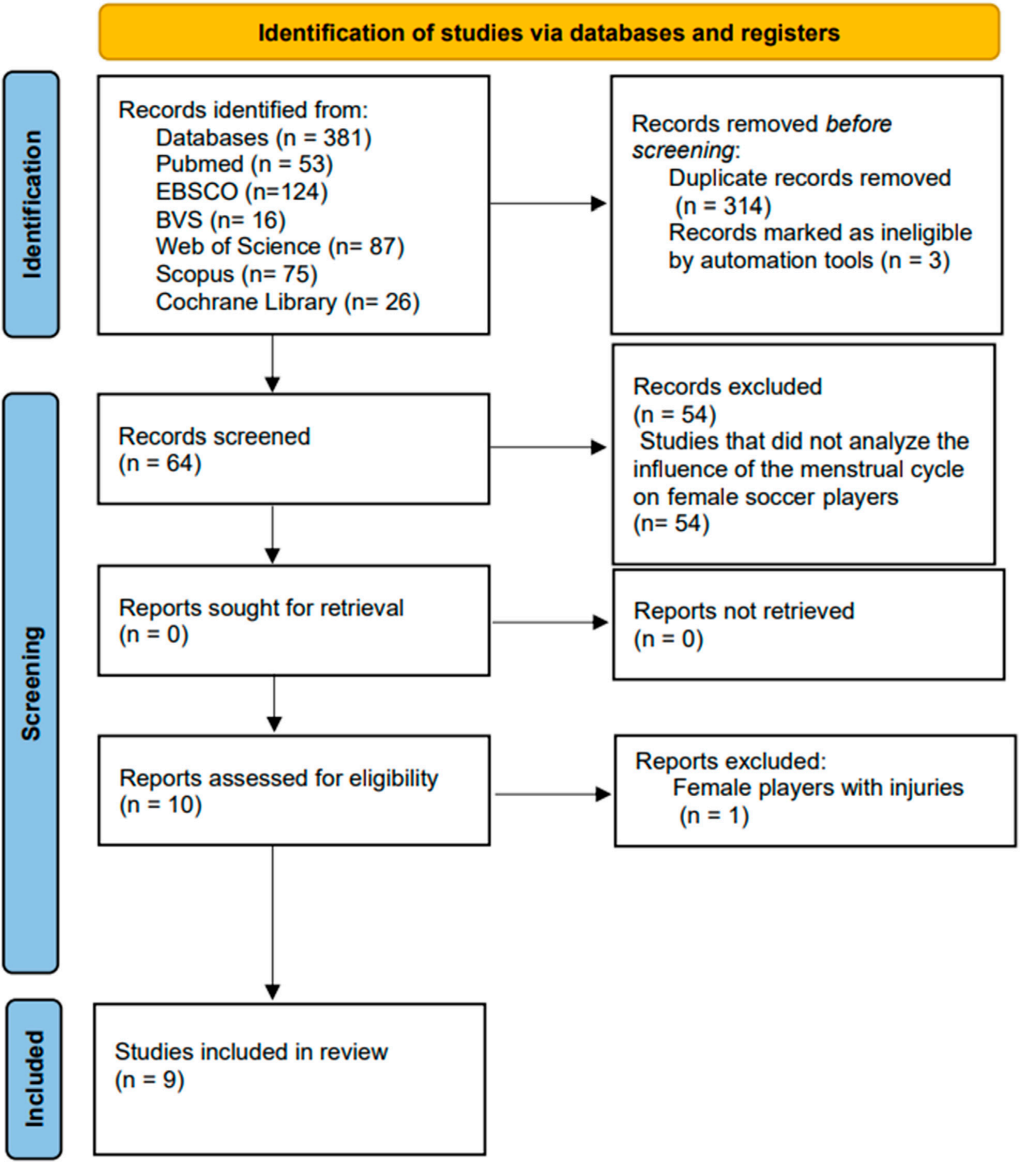
Flow diagram illustrating the study selection process.

The following flow diagram illustrates the study selection process (Figure 1).

### 3.2 General characteristics of the selected studies

The studies included in this systematic review were randomized controlled clinical trials, published between 2009 and 2022 (MENSTRUAL, 2009; Tsampoukos et al., 2010; Julian et al., 2017; Vila, 2017; Tounsi et al., 2018; Carmichael et al., 2021; Nabo et al., 2021; Campa et al., 2022; Igonin et al., 2022). It should be noted that with the exception of one study from 2009 (Julian et al., 2017) all the others can be considered current as women’s sport has only been on the rise for a few years.

Of the 9 studies, 3 were developed in Spain (MENSTRUAL, 2009; Tsampoukos et al., 2010; Vila, 2017), 2 in Germany (Julian et al., 2017; Carmichael et al., 2021), 1 in Canada (Tounsi et al., 2018), 1 in Portugal (Nabo et al., 2021), 1 in Italy (Campa et al., 2022) and the remaining in France (Igonin et al., 2022).

The sum of the initial sample size, consisting of the nine included articles, groups together a total of 256 individuals, while the sum of the final sample, consisting of all the studies, groups together a total of 117 studies. There were no adverse effects in relation to the application of the performance tests. The losses observed in the sample were due to exclusion criteria that had been established before starting the tests and included injured players, players who took the contraceptive pill and others who had irregular periods.

In relation to gender, the entire study population was elite and sub-elite female footballers. Moreover, the age range was quite homogeneous and limited in all the clinical trials, being within the 16–30 years of age range.

Below, the characteristics of the studies and the sample as shown in Table 1.

**TABLE 1:** Characteristics of the included studies.

Author	Year	Country	Sample1	Exclusion criteria	Sample2	Category	Height	Body Mass	Age
Guijarro E et al. (MENSTRUAL, 2009)	2009	Spain	N = 16	-	N = 16	1ºDIV	-	-	22.87
Villa del Bosque et al. (Vila, 2017)	2017	Spain	N = 19	Injury or indisposition	N = 12	2ºDIV	163.00 ± 0,0436 m	57.82 ± 1.35 kg	19.63 ± 2.34
Julian R et al. A (Julian et al., 2017)	2017	Germany	N = 35	Contraceptives, irregular periods, less than 5 years participating in official competitions	N = 9	2º Female League	161.3 ± 6.6	Follicular phase:59.1 ± 7.7/Luteal phase:58.8 ± 7.5	19 ± 4
Tounsi M et al. (Tounsi et al., 2018)	2018	Canada	N = 11	-	N = 11	High level	1.63 ± 0.05 m	59.02 ± 7.59 kg	21.18 ± 3.15
Julian R et al. B (Carmichael et al., 2021)	2020	Germany	N = 83	Girls with regular cycle of biological condition, contraceptives, having ovulatory cycles	N = 15	1° y 2° DIV	-	-	-
Nabo et al. (Nabo et al., 2021)	2020	Portugal	N = 14	-	N = 14	1° DIV	1.64 ± 0.06 m	59.6 ± 9.1 kg	24.1 ± 4.1
Campo F et al. (Campa et al., 2022)	2021	Italy	N = 20	-	N = 20	1° DIV	1.63 m	61.4 kg	23.8
Sánchez M et al. (Tsampoukos et al., 2010)	2021	Spain	N = 16	Menstrual cycle variation	N = 12	Regional	164.01 ± 0,0727 m	61.90 ± 6.37 kg	16.18 ± 1.68
Igonin P et al. (Igonin et al., 2022)	2022	France	N = 42	Contraceptives, goalkeeper, participants who did not play at least 3 games in each phase of their cycle	N = 8	2ºDIV	167.3 ± 7.2	58.9 ± 6.3	25.7 ± 3.3

**Abbreviations**: DIV: soccer division category, m:meters; kg:kilograms.

### 3.3 Risk of bias in the included studies

Regarding the assessment of bias risk in the studies, all articles (MENSTRUAL, 2009; Tsampoukos et al., 2010; Julian et al., 2017; Vila, 2017; Tounsi et al., 2018; Carmichael et al., 2021; Nabo et al., 2021; Campa et al., 2022; Igonin et al., 2022) were at a high risk of bias in at least one field. All studies showed a low risk of bias for incomplete results data and in selective reporting of results.

Moreover, adequate sequence generation and sequence concealment showed a high risk of bias for all studies.

Blinding of participants and staff indicated an unclear risk of bias.

Table 2 shows the risk of bias for the articles included. The different colors shown in the table present the methodological quality of the studies: High risk (red), unclear risk (yellow), low risk of bias (green).

**TABLE 2:** Risk of bias.

	Guijarro E et al. (MENSTRUAL, 2009)	Villa del Bosque et al. (Vila, 2017)	Julian R et al.A (Julian et al., 2017)	Tounsi M et al. (Tounsi et al., 2018)	Julian R et al.B (Carmichael et al., 2021)	Nabo et al. (Nabo et al., 2021)	Campa F et al. (Campa et al., 2022)	Sánchez M et al. (Tsampoukos et al., 2010)	Igonin P et al. (Igonin et al., 2022)
Appropriate sequence generation (selection risk)	−	−	−	−	−	−	−	−	−
Selection hiding (selection bias)	−	−	−	−	−	−	−	−	−
Blinding of the participants and staff (implementation bias)	?	?	?	?	?	?	?	?	?
Blinding of the outcome assessors (detection bias)	?	?	?	?	?	?	?	?	?
Incomplete results data (attrition bias)	+	+	+	+	+	+	+	+	+
Selective reporting of results (notification bias)	+	+	+	+	+	+	+	+	+
Other sources of bias	+	+	+	+	+	+	+	+	+

Abbreviations: (+): Low risk of bias (−): High risk of bias (?): Unclear risk of bias.

### 3.4 Intervention characteristics

All participants in all studies were characterized by having regular menstruation and not taking birth control pills.

The menstrual cycle controls were carried out in different ways depending on the study. In 5 studies (MENSTRUAL, 2009; Tsampoukos et al., 2010; Vila, 2017; Nabo et al., 2021; Igonin et al., 2022) the calendar method was used, where the players wrote down their menstrual cycles, including previous cycles. One study used a menstruation diary (Julian et al., 2017) where the players recorded the phases of the cycle in which they were. In two other studies hormone levels were measured through blood tests (Tounsi et al., 2018; Carmichael et al., 2021) and the remaining study used self-determination (Campa et al., 2022).

Performance was the variable being studied. In all the studies tests were carried out in the different phases of the menstrual cycle to compare the results and observe if differences were established between each phase. In these studies (MENSTRUAL, 2009; Tsampoukos et al., 2010; Tounsi et al., 2018) a warm-up was performed prior to the tests.

1. In the study by Guijarro et al. (MENSTRUAL, 2009) the “Course Navette” test was used to assess physical performance; it consisted of a maximum test where the demands of oxygen consumption are important. In the test a player covers a distance of 20 m as many times as possible at a progressive intensity of work. In this study, the perception of effort of each player was measured with the CR-10 effort perception scale.
2. In the Villa del Bosque study (Vila, 2017) a 30-m speed test using photoelectric cells was used to assess performance.
3. In the study by Julian R et al. A (Julian et al., 2017) the following tests were used for performance assessment: the YO-YO intermittent endurance test (IET), jumps and sprint 3 × 30 m.
4. In the study by Tounsi M et al. (Tounsi et al., 2018) the 5-jump test was used to evaluate the explosive power of lower limbs in soccer players, the sprint-shuttle test consisting of 6 round trip sprints of 40 m with changes of direction to assess skill and the YO-YO intermittent recovery test Level 1, which consisted of performing round trips of 2 × 20 m at increasing speeds.
5. Julián R et al.B (Carmichael et al., 2021), assessed performance in matches using a 5 Hz (HZ) GPS device that was placed on the players’ ankles. The players had to play at least 75 min in matches in each of the phases of the menstrual cycle.
6. Nabo et al. (Nabo et al., 2021) used a calibrated treadmill to perform a cardiorespiratory test where the footballer was required to reach fatigue or maximum heart rate. The test was carried out in the presence of a doctor and an emergency team.
7. Campa et al. (Campa et al., 2022) performed the 20-m sprint and jump test with test assessments being performed on the second day of each early follicular phase and 14 days later when participants were in their ovulatory phase.
8. In the study by Sánchez et al. (Tsampoukos et al., 2010) three tests were performed. The tests were: 1) the 40-m test where the maximum linear speed was determined in two linear sprints of 40 m separated by a passive break of 2 minutes; the best attempt of the two sprints was selected for analysis, 2) the V-Cut test to determine the change of direction speed, where 2 sprints of 25 m were carried out with four changes of direction and 2 minutes of passive rest between each sprint, and 3) the horizontal jump test where the monopodal jump, to establish the dominant leg, and the bipodal jump were evaluated.
9. In the article by Igonin et al. (Igonin et al., 2022) the movements of each woman in competition matches for 3 seasons were recorded to determine if a player’s performance was affected by any phase of the menstrual cycle.

All these described tests that have been used in the included studies assess the aerobic capacity of female soccer players in the Follicular and Luteal phases of the menstrual cycle, aiming to evaluate whether fatigue and effort increase during any of these phases or if the athlete’s recovery time is longer, thereby influencing sports performance.

The duration of the intervention varied from 7 weeks (Vila, 2017; Campa et al., 2022), 8 weeks (Julian et al., 2017), 12 weeks (Tsampoukos et al., 2010) and 16 weeks (MENSTRUAL, 2009; Carmichael et al., 2021) to 156 weeks (Igonin et al., 2022).

Table 3 shows the intervention characteristics in detail.

**TABLE 3:** Intervention characteristics.

Author	Year	Intervention	Intervention duration (weeks)	Menstrual cycle control	Variables	Measuring instrument
MENSTRUAL (2009)	2009	course navette	16	calendar	performance	measuring tape
Vila (2017)	2017	2 sprints max 30 m	7	calendar	performance	photoelectric cells
Julian et al. (2017)	2017	jump, 3 × 30 M races	8	menstruation diary	performance	
		YO-YO Intermittent endurance				
Tounsi et al. (2018)	2018	5 jumps, sprint ability and the YO-YO intermittent recovry test level 1		serum progesterone levels	performance	measuring tape
Carmichael et al. (2021)	2020	The average speed for the distance covereds	16	Blood and urine analysis and period days logged by mail	performance	gps device
Nabo et al. (2021)	2020	Maximum cardiorespiratory endurance test	-	calendar	cardiorespiratory performance	electronic hr monitor
Campa et al. (2022)	2021	20 M spriint and jump	7	self determined	performance	shapiro wilk test
Tsampoukos et al. (2010)	2021	40 m, v-cut and horizontal jump	12	calendar	performance	hooper scale
Igonin et al. (2022)	2022	sprint and Distance Covered	156	calendar	performnace	inertial measuring unit on the player’s ankle

Abbreviations: MAX: maximum; M:metres, HR:heart rate.

### 3.5 Results

This systematic review consists of nine trials, in which there is a great disparity in the results. These results can be divided into 2 main ideas.

In five studies (Tsampoukos et al., 2010; Vila, 2017; Tounsi et al., 2018; Carmichael et al., 2021; Campa et al., 2022) no changes were observed in the performance of the soccer players in the different phases of the menstrual cycle.

However, in 4 studies significant changes were found in the performance of female footballers in the phases of the menstrual cycle, although not all studies agreed on the results.

In the cases Guijarro et al. (Vila, 2017) and Igonin et al. (Igonin et al., 2022) negative changes were observed in performance in the follicular phase.

The study by Nabo et al. (Nabo et al., 2021) focuses on an increase in cardiorespiratory performance in the luteal phase compared to the follicular phase of the menstrual cycle, although the authors state that there is still much controversy in the existing literature about the relationship between hormonal changes in the menstrual cycle and physical exercise.

On the other hand, in the study by Julián R et al. (Julian et al., 2017) there is a reduction in endurance performance during the luteal phase of the menstrual cycle, however the same findings were not found in other sprint and jump performance tests, so the authors do not think that this change is associated with the menstrual cycle phase.

### 3.6 Grade system

The evidence quality in this systematic review is very low. Assessments have relied heavily on the risk of bias of the trials and the imprecision of their results, mainly due to disparity in sample sizes.

For more detailed information, see Supplementary Table S5.

## 4 Discussion

This systematic review was carried out with the aim of analyzing whether performance is influenced by any of the phases of the menstrual cycle in female footballers. There is very low-quality evidence in all studies and there is a great deal of controversy in our results.

This review consists of nine controlled clinical trials (MENSTRUAL, 2009; Tsampoukos et al., 2010; Julian et al., 2017; Vila, 2017; Tounsi et al., 2018; Carmichael et al., 2021; Nabo et al., 2021; Campa et al., 2022; Igonin et al., 2022). The results have been organized into two main ideas.

In the studies by Villa del Bosque (Vila, 2017), Tounsi M et al. (Tounsi et al., 2018), Julian R et al. B (Carmichael et al., 2021), Campa F et al. (Campa et al., 2022) and Sánchez M et al. (Tsampoukos et al., 2010) no differences were found in the performance of female football players between phases of the menstrual cycle. These findings coincide with the results of the study by Vaiksaar et al. (Vaiksaar et al., 2011) also found no difference in sports training in female rowers in the different phases of the menstrual cycle. On the other hand, another study on elite athletes indicates that the menstrual cycle phases do affect the performance of the players (Carmichael et al., 2021).

On the other hand, we find the studies of Guijarro et al. (MENSTRUAL, 2009), Julián et al. A (Julian et al., 2017), Nabo et al. (Nabo et al., 2021) and Igonin et al. (Igonin et al., 2022) where there were changes in performance in relation to the phases of the menstrual cycle.

In the studies by Guijarro et al. (MENSTRUAL, 2009) and Igonin et al. (Igonin et al., 2022) the authors considered that there was a negative effect on movement patterns and performance in the follicular phase compared to the other phases of the menstrual cycle. In the study by Gimenez-Blasi et al. (Giménez-blasi et al., 2022) it was stressed that performance can be influenced in the early follicular phase, and can be improved in the other phases of the menstrual cycle. These results are consistent with a meta-analysis published in 2020 (McNulty et al., 2020).

These effects may be due to the fact that in the follicular phase there is estrogen production, which plays an important role throughout the menstrual cycle, having a neuro-excitatory effect that may involve changes in sports performance (Brown et al., 2021).

In the study by Nabo et al. (Nabo et al., 2021) an increase in cardiorespiratory performance in the luteal phase is seen, although no significant changes are found in the other performance tests, so the authors think that these changes were not due to physiological variations caused by the menstrual cycle.

Finally, in the study by Julian R et al. A (Julian et al., 2017) there is a decrease in endurance performance during the luteal phase compared to the follicular phase of the menstrual cycle. These findings are consistent with the results of a study that recognized that the menstrual cycle causes physical symptoms that develop during the luteal phase and disappear within a few days of menstruation (Brown et al., 2021).

This could be explained inasmuch as the physiological response to exercise can be influenced by endocrine hormones that are produced in the phase of the menstrual cycle: during the luteal phase there is an increase in progesterone which is associated with a greater degree of effort, which may be related to a decrease in performance (Reilly, 2000).

The disparity of results given in the 9 included studies is not surprising at all. First, the primary outcome is different in most of the included studies. Sprint performance demands complete other conditions than endurance and overall capacity. Differences between conditions such as endurance, speed, coordination and even decision making are a result of muscle mass, muscle strength, muscle composition, peripheral neural innervation, neuro-muscular connections, tendon and ligament structure, the gut microbiome, collagen quality and central motor system anatomy and functioning, next to others (Furrer et al., 2023). Estrogens further show neuro-excitatory effects that may involve changes in sports performance (Paludo et al., 2022).

**FIGURE 2:**
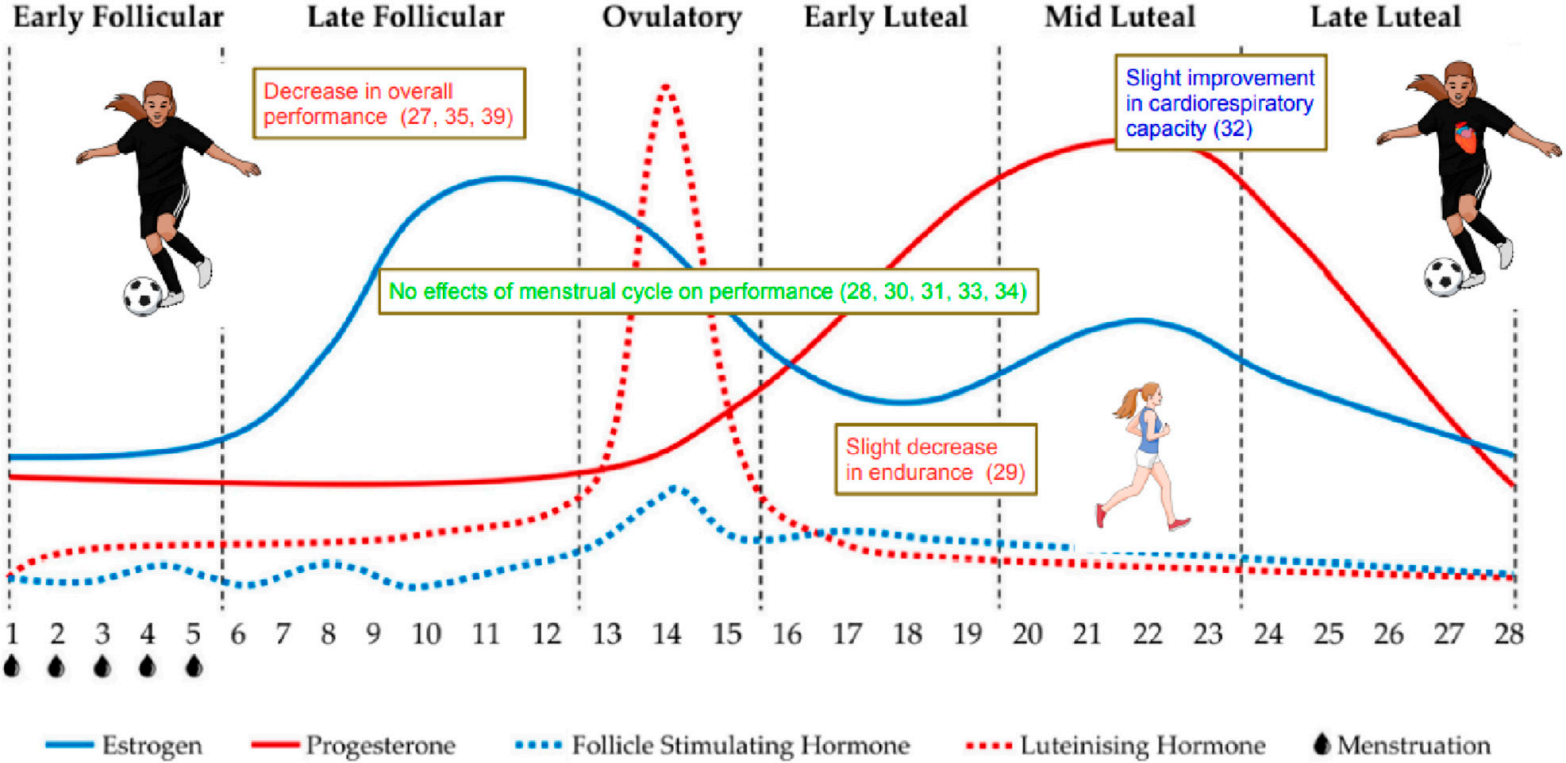
An overview of the different menstrual cycle phases and the results of the systematic review.

All these parameters are interconnected but are differently influenced by sex hormones and other signaling substances such as cytokines and neurotransmitters; substances that differ between men and women in both quantity and sensitivity. High levels of estrogens, for instance, are related with increased bone and muscle function, it decreases resistance of tendons and ligaments (Chidi-Ogbolu and Baar, 2019). The opposite effects of estrogens on muscle and bone on one side and tendons and ligaments on the other side could provide an explanation for the difference of the influence of the menstrual cycle on performance. Another factor that can influence the impact of higher or lower hormone levels and performance female football players is the possible impact of nutrition on hormone level, function and performance. High intake of soy, as food or supplement, affects different parameters related with performance in general and sprint capacity specifically. A recent systematic review found that the use of soy supplements could improve antioxidative capacity, enhance speed and increase speed resistance during repeated bouts of high intensity exercises (Zare et al., 2023). Figure 2 summarizes the menstrual cycle phases and the results of our systematic review.

Note that results are very dispaired and no general recommendations or conclusions can be drawn. Whereas estrogens during the follicular phase tend to decrease overall performance, other studies cited in our review conclude the opposite. The luteal phase, characterized by high progesterone seems to enhance cardiovascular capacity (Nabo et al., 2021), although the results of another study suggests the opposite (Julian et al., 2017). As concluded, hormonal influences on performance seem only minor in women playing football. Social factors and gender are probably responsible for the absence of influence and therefore integrative studies combining both biological and social factors are needed desperately. This is also based on the fact that female soccer is booming and support on performance improvement and injury prevention are essential for the further development of female football.

The most common comparison found in the literature when investigating the effects of the menstrual cycle on athlete performance is between the early follicular phase and the luteal phase; this is due to the fact that the hormonal difference is maximum in these two phases (Giménez-blasi et al., 2022). The changes in female steroid hormones (estrogen and progesterone), which happen during ovulatory menstrual cycles, have many physiological effects that could influence physical exercise. In the follicular phase estrogen release occurs while in the luteal phase large amounts of estrogen and progesterone are secreted (Janse et al., 2019). In a study by Brunivels et al. (Bruinvels et al., 2016), 51% of athletes perceived that the menstrual cycle affected their performance.

The study by Iersel KC et al. reveals that in adolescents there is a strong association between physical and psychological symptoms (van Iersel et al., 2016), therefore, hormonal changes that affect women psychologically during menstruation can also influence their performance. These psychological changes (low mood, stress, etc.) that occur during menstruation can be a consequence of cortisol hormone release and variations in the level of cytokines, with cytokine IL-6 being the cytokine most studied during exercise. These emotional changes can compromise the physical abilities of the players altering their performance; hence, it can be seen that a good performance requires a good emotional balance (Foster et al., 2019).

In short, changes during the menstrual cycle may affect exercise performance, and therefore should be taken into account during sports training (Pallavi et al., 2017). The disparity in the results may be due to the fact that the most optimal tests for assessing the menstrual cycle are not yet established (Pallavi et al., 2017). We found a systematic review where contradictory results are also obtained (Vescovi and Favero, 2014).

Several reasons for the ambiguous results between studies included in this systematic review vary from psycho-social factors, via early life exposure to testosterone and, as mentioned earlier, nutritional aspects. Gender differences have long be considered the same as biological differences between men and women. Actually gender and sex are considered two different factors when comparing men and women. The world health association defines sex and biology as follows:

“The World Health Organisation summarises the difference between sex and gender in the following way: Sex refers to “the different biological and physiological characteristics of males and females, such as reproductive organs, chromosomes, hormones, etc.”

Gender refers to “the socially constructed characteristics of women and men—such as norms, roles and relationships of and between groups of women and men. It varies from society to society and can be changed. The concept of gender includes five important elements: relational, hierarchical, historical, contextual and institutional. While most people are born either male or female, they are taught appropriate norms and behaviours—including how they should interact with others of the same or opposite sex within households, communities and work places. When individuals or groups do not “fit” established gender norms they often face stigma, discriminatory practices or social exclusion—all of which adversely affect health”.

Disparity between results could mean that the biological impact of the menstrual cycle is less great than imagined. Perhaps gender impact is greater than ever thought. Women who engage in top sports are probably highly motivated and focused on success. Women who choose for a more ‘masculine’ social role show significantly higher testosterone levels than women with higher self-reported femininity (van Anders et al., 2015). Higher testosterone levels could increase performance in women, although a new study in 2023 (Bezuglov et al., 2023) does not support this conclusion.

Prenatal testosterone levels could also confound the impact of the different phases of the menstrual cycle on performance. Both the digit-ratio (2D:4D) and the anogenital distance indicate the level of testosterone exposure prenatal and early postnatal in male and female children (Zheng and Cohn, 2011; Crespi and Evans, 2023). Lower D2:D4 Digit and lower anogenital distance indicate higher testosterone exposure in early life, and higher early life exposure to testosterone is related with less pain sensitivity and premenstrual symptoms (Crespi and Evans, 2023) and greater success rate in Olympic sports (Eklund et al., 2020).

Aerobic and anaerobic performance, both important necessities in football, are differently influenced by hormones and so can theoretically menstrual cycle phases. Two extensive meta-analysis and systematic reviews investigated menstrual cycle of performance in different sports types (Janse et al., 2019; Findlay et al., 2020). Both studies conclude that there are only very slight differences in aerobic and anaerobic performance in different menstrual cycle phases. The included studies in both reviews did not produce enough evidence to provide evidence-based recommendations, because of trivial effect, low coherence in study design and low study quality (Janse et al., 2019; Blagrove et al., 2020).

Early testosterone exposure, gender role and nutritional factors could overrule or even change the possible impact of sex hormones on performance, although integrative studies that would include all the mentioned factors have to be developed.

### 4.1 Practical application

It is still not clear whether the hormonal changes of menstruation influence the physical performance of female footballers, yet it has been detected that these changes worsen the psychological wellbeing of athletes, which could be related to a decrease in performance although study results are contradictory. Consequently, it would be of great interest to continue doing research on this issue.

### 4.2 Study limitations and strengths

Our study was limited by the scarce scientific evidence on the influence of menstruation on the performance of soccer players. The strength of our study is reflected in the disparity of results. Those results show that the biological impact of hormones on performance in female football players is overrated and this itself opens new research windows with less biological bias and more focus on gender as a possible determinant factor in female sports.

## 5 Conclusion

This systematic review shows that there is a great deal of controversy on the menstrual cycle phases influencing the performance of female footballers. Although several studies do find impact of menstrual cycle phase on performance, others do not support these findings. As discussed, many factors next to the monthly period, influence performance both directly and indirectly, confounding functional influences of hormonal changes during the menstrual cycle. The disparity should only be interpreted as positive, because it opens up the window for non-biased research on female football players worldwide. Female football is in its booming moment and therefore further studies in this line are needed to suit this moment in the general society and the scientific world.

## Data Availability

The original contributions presented in the study are included in the article/Supplementary Material, further inquiries can be directed to the corresponding author.

## Abbreviations

DIV: soccer division category; M: Meters; Kg: Kilograms; YO-YO intermittent endurance test (IET); Hz: Hertz; Max: Maximum; HR: Heart Rate.
