# Supplementary material for: Effects of the menstrual cycle on the performance of female football players. A systematic review
Source: Front Physiol. 2024 Apr 8;15:1359953. doi: 10.3389/fphys.2024.1359953 (PMC11033481; doi:10.3389/fphys.2024.1359953)
Supplement: Supplementary file 1 [file Table1.pdf]

Supplementary file 1: Search Strategies.

Table S1. Search strategies

Platform	Search strategies
PubMed	(("female soccer players"[tw]) AND ("Menstrual Cycle"[Mesh] OR "Luteal Phase"[Mesh] OR "Follicular Phase"[Mesh]) AND ("Athletic Performance"[Mesh])) OR ((“female soccer players”[tw]) AND (“Menstrual cycle” [tw] OR “Period” [tw]) AND (“performance”[tw] OR “Athletic performance”[tw]))
SCOPUS	(("female soccer players”) AND ("Menstrual Cycle” OR "Luteal Phase" OR "Follicular Phase") AND ("Athletic Performance")) OR ((“female soccer players”) AND (“Menstrual cycle” OR “Period”) AND (“performance” OR “Athletic performance”))

Table S1. Search strategies

Platform	Search strategies
BVS	(tw:("female soccer players”) AND Mh:"Menstrual Cycle” OR "Luteal Phase" OR "Follicular Phase") AND Mh:("Athletic Performance")) OR tw:((“female soccer players”) AND (“Menstrual cycle” OR “Period”) AND (“performance” OR “Athletic performance”))
Web of science	TS=((“female soccer players”) AND (“Menstrual cycle” OR “Period”) AND (“performance” OR “Athletic performance”))

Table S1. Search strategies

**Platform**	**Search strategies**		
EBSCO	(("female soccer players”) AND MH("Menstrual Cycle” OR "Luteal Phase" OR "Follicular Phase") AND MH("Athletic Performance")) OR ((“female soccer players”) AND (“Menstrual cycle” OR “Period”) AND (“performance” OR “Athletic performance”))		
Cochrane library	#1	female soccer players	287
	#2	MeSH descriptor: [Menstrual Cycle] explode all trees	2004
	#3	MeSH descriptor: [Luteal Phase] explode all trees	564
	#4	MeSH descriptor: [Athletic Performance] explode all trees	8079
	#5	menstrual cycle	6089
	#6	Period	269012
	#7	performance	123835
	#8	athletic performance	3823
	#9	((#1) AND (#2 OR #3) AND (#4)) OR ((#1) AND (#5 OR #6) AND (#7 OR #8))	26

Supplementary file 2: GRADE System.

Table S5: GRADE System.

Author(s): Dina Hamed-Hamed, Ana González-Muñoz, Maria Cuevas-Cervera, Jose Javier Perez-Montilla, Daniel Aguilar-Nuñez, María Aguilar-García, Leo Pruimboom, Santiago Navarro-Ledesma.

Question: Influence of menstruation compared to performance for women soccer players

Setting: Physiotherapy

Bibliography:

Certainty assessment							Impact	Certainty	Importance
№ of studies	Study design	Risk of bias	Inconsistency	Indirectness	Imprecision	Other considerations			
**New outcome**									
9	randomised trials	serious[a]	serious[b]	not serious	very serious[c]	dose response gradient	Four studies showed changes in performance during the phases of the menstrual cycle, while the remaining five stufies showed no oscillations in performance	⨁◯◯◯ Very low	IMPORTANT

CI: confidence interval

Explanations

a. Due to the lack of sequence concealment in all studies and the unclear risk of blinding of participants and staff, it is not clear whether the studies were conducted in a blinded manner.

b. Four studies had significant differences, while 5 studies had no significant differences.

c. Due to the sample analysis analyzed (<400)
